# Does Antimicrobial Therapy Affect Mortality of Patients with Carbapenem-Resistant *Klebsiella pneumoniae* Bacteriuria? A Nationwide Multicenter Study in Taiwan

**DOI:** 10.3390/microorganisms8122035

**Published:** 2020-12-19

**Authors:** Chien Chuang, Chin-Fang Su, Jung-Chung Lin, Po-Liang Lu, Ching-Tai Huang, Jann-Tay Wang, Yin-Ching Chuang, L. Kristopher Siu, Chang-Phone Fung, Yi-Tsung Lin

**Affiliations:** 1 Division of Infectious Diseases, Department of Medicine, Taipei Veterans General Hospital, 11217 Taipei, Taiwan; s19601031@gm.ym.edu.tw; 2 Division of Infectious Diseases, Department of Internal Medicine, Chia-yi Branch, Taichung Veterans General Hospital, Chiayi 60090, Taiwan; 3 Division of Allergy, Immunology and Rheumatology, Department of Medicine, Taipei Veterans General Hospital, Taipei 11217, Taiwan; cfsu@vghtpe.gov.tw; 4 Division of Infectious Diseases and Tropical Medicine, Department of Internal Medicine, Tri-Service General Hospital, National Defense Medical Center, Taipei 11490, Taiwan; jclin@ndmctsgh.edu.tw; 5 Department of Internal Medicine, Kaohsiung Medical University Hospital, Kaohsiung 80756, Taiwan; idpaul@gmail.com; 6 College of Medicine, Kaohsiung Medical University, Kaohsiung 80756, Taiwan; 7 Division of Infectious Diseases, Department of Internal Medicine, Linkou Chang Gung Memorial Hospital, Taoyuan 33305, Taiwan; chingtaihuang@gmail.com; 8 Division of Infectious Diseases, Department of Medicine, National Taiwan University Hospital, Taipei 10048, Taiwan; wang.jt1968@gmail.com; 9 Department of Internal Medicine and Medical Research, Chi Mei Medical Center, Tainan 71004, Taiwan; chuangkenneth@hotmail.com; 10 Institute of Infectious Diseases and Vaccinology, National Health Research Institutes, Miaoli 35053, Taiwan; klksiu@gmail.com; 11 Division of Infectious Diseases, Sijhih Cathay General Hospital, New Taipei City 10630, Taiwan; cpfung2000@gmail.com; 12 Institute of Emergency and Critical Care Medicine, National Yang-Ming University, Taipei 11221, Taiwan

**Keywords:** carbapenem resistant, *Klebsiella pneumoniae*, bacteriuria, antimicrobial therapy, critically ill

## Abstract

Few clinical studies have previously discussed patients with carbapenem-resistant *Klebsiella pneumoniae* (CRKP) bacteriuria. This study aimed to assess the effect of antimicrobial therapy on the mortality of patients with CRKP bacteriuria. Hospitalized adults with CRKP bacteriuria were enrolled retrospectively from 16 hospitals in Taiwan during 2013 and 2014. Critically ill patients were defined as those with an Acute Physiology and Chronic Health Evaluation (APACHE) II score ≥ 20. Multivariate Cox regression analysis was used to determine independent risk factors for 14- and 28-day mortality. Of 107 patients with CRKP bacteriuria, the 14-day and 28-day mortality was 14.0% and 25.2%, respectively. Thirty-three patients received appropriate antimicrobial therapy. In the multivariate Cox regression analysis, the APACHE II score ≥ 20 was the only independent risk factor for 14-day mortality (hazard ratio [HR]: 6.15, *p* = 0.024). APACHE II score ≥ 20 (HR: 3.05, *p* = 0.018) and male sex (HR: 2.57, *p* = 0.037) were associated with 28-day mortality. Among critically ill patients with CRKP bacteriuria, appropriate antimicrobial therapy was not associated with 14-day or 28-day survival. In conclusion, in patients with CRKP bacteriuria, the use of appropriate antimicrobial therapy was not an independent factor associated with reduced mortality. Our findings may inform future antibiotic stewardship interventions for bacteriuria caused by multidrug resistant pathogens.

## 1. Introduction

Carbapenem-resistant *Klebsiella pneumoniae* (CRKP) has been increasingly reported in both healthcare associated infection and environment during recent years worldwide [1,2,3]. Treating CRKP infection is challenging due to its high mortality [4,5,6,7,8] and the limited therapeutic options available [9,10,11]. The most common mechanism of CRKP is carbapenemase production, followed by combinations of decreased membrane permeability and overexpression of extended spectrum β-lactamase (ESBL) or AmpC β–lactamase [12,13].

CRKP has frequently been isolated from the urine of hospitalized patients with CRKP infection or colonization [14,15]. However, clinical studies regarding CRKP bacteriuria are limited [16,17,18,19,20,21,22]. The 30-day mortality of patients with CRKP bacteriuria, with or without infection, is between 6% and 16% [16,18]. One recent study showed that 30-day mortality of patients with CRKP urinary tract infection was 23.2% [22]. Qureshi et al. found that up to 80% of patients with CRKP bacteriuria were asymptomatic bacteriuria and it did not lead to subsequent infections or death [18]. However, many patients with CRKP bacteriuria receive antimicrobial therapy because differentiating colonization from infection is difficult in clinical practice [16,21]. Aminoglycosides had been demonstrated to have higher microbiologic clearance of CRKP bacteriuria than polymyxin B or tigecycline [16]. Another study also found that treatment with aminoglycoside was associated with a higher rate of clinical success among patients with CRKP bacteriuria, but treatment with tigecycline was associated with a higher failure rate [21]. Nonetheless, all the aforementioned studies did not investigate the risk factors of mortality in patients with CRKP bacteriuria. Furthermore, 13–20% CRKP bacteriuria occurs in critically ill patients [16,17,21]. However, the characteristics and clinical outcomes of critically ill patients with CRKP bacteriuria have not been studied previously. 

This study aimed to determine factors associated with fatal outcomes in patients with CRKP bacteriuria of varying degrees of severity. We focused on the effect of antimicrobial therapy on mortality in patients with CRKP bacteriuria.

## 2. Materials and Methods

### 2.1. Participants and Study Design

We conducted this retrospective study at 16 hospitals (12 medical centers and 4 regional/local hospitals) in Taiwan from January 2013 to December 2014. Electronic medical records of hospitalized patients with CRKP bacteriuria were reviewed. Patients with polymicrobial pathogens in their urine, those aged < 20 years, and those who died within 48 h after the index culture, were excluded. Patients who did not receive any antimicrobial therapy were not included for outcome analysis.

The study protocol was approved by the ethics review boards of each participating hospital. Patient consent was not required because the study was a retrospective review of patient medical records.

### 2.2. Definitions and Outcomes

Hospital-acquired bacteriuria was defined as bacteriuria in an index culture collected >48 h after the patient’s admission to hospital. The definition of healthcare-associated bacteriuria was defined as community-onset events with previous exposure to healthcare facilities as described previously [23]. An immunocompromised state was defined as the presence of neutropenia, human immunodeficiency virus infection, steroid therapy (≥20 mg of prednisone or equivalent per day for ≥1 month), or other immunosuppressive therapy during the 30 days preceding the index culture. The severity of illness at the onset of infection was graded using the highest acute physiology and chronic health evaluation (APACHE) II score within 48 h, before or after, the index culture. Critically ill patients were defined as those with an APACHE II score ≥ 20. Shock was defined as patients needing a vasopressor to maintain the mean arterial pressure ≥65 mmHg despite the administration of fluid resuscitation within 48 h of the index culture. Appropriate antimicrobial therapy was defined as having been treated with at least one antimicrobial agent in vitro active against CRKP for ≥48 h, initiated within 7 days after the index culture [16,21]. Patients who received tigecycline as the only antimicrobial therapy were also defined as inappropriate therapy [22]. Patients treated with any regimen that contained an aminoglycoside were classified as having received aminoglycoside-based therapy. Patients treated with any regimen that contained colistin, but not an aminoglycoside, were classified as having received colistin-based therapy [21]. Early appropriate antimicrobial therapy was defined as having received an effective antimicrobial agent for CRKP within 5 days after onset of infection, with effectiveness determined by the sensitivity test done in vitro. The primary outcomes were 14- and 28-day mortality.

### 2.3. Bacterial Isolates

During the study period, CRKP clinical isolates were collected from each participating hospital. These isolates were then sent to National Health Research Institutes (NHRI) in Miaoli, Taiwan, and stored at −70 °C in 10% glycerol Luria-Bertani medium before analysis. Carbapenem resistance was defined as a minimal inhibitory concentration (MIC) ≥ 2 μg/mL for imipenem or meropenem [24]. Only the first episode of CRKP bacteriuria in each patient was included for analysis. All CRKP clinical isolates were identified by the microbiological laboratories of each participating hospital, and confirmed with Vitek 2 automated system (bioMérieux, Marcy l’Étoile, France) in NHRI.

### 2.4. Antimicrobial Susceptibility Testing

Broth microdilution was used to determine MICs (Sensititre, Trek Diagnostic Systems, Cleveland, OH, USA) for all antibiotics, except tigecycline. The CLSI M100-S22 Performance Standards were applied for interpreting MIC results for all antimicrobial agents, except tigecycline and colistin [24]. Tigecycline MICs were tested by E-test (AB Biodisk, Solna, Sweden) on Mueller-Hinton media, and classified according to the breakpoints established by the Food and Drug Administration criteria (≤2.0 mg/L, susceptible; 4.0 mg/L, intermediate; and ≥8.0 mg/L, resistant). The susceptibility to colistin was interpreted based on the European Committee on Antimicrobial Susceptibility Testing [25]. Quality control was performed using *Escherichia coli* ATCC 29212, and *Pseudomonas aeruginosa* ATCC 27853.

### 2.5. Detection of Genes Encoding for Carbapenemase, AmpC β-Lactamase, and ESBLs

Polymerase chain reaction was performed to detect carbapenemase genes (Ambler class A families *bla*_KPC_, *bla*_NMC_, *bla*_IMI_, *bla*_SME_, and *bla*_GES_; Ambler class B families *bla*_IMP_, *bla*_VIM_, *bla*_NDM_, *bla*_GIM_, *bla*_SPM_, and *bla*_SIM_; and Ambler class D family *bla*_OXA-48_), plasmid-borne AmpC-like genes (*bla*_CMY_, and *bla*_DHA_), and ESBL genes (*bla*_CTX-M_, *bla*_TEM_, and *bla*_SHV_), with the primers listed previously [26]. For *bla*_SHV_, *bla*_CTX-M_, *bla*_TEM_, *bla*_CMY_, *bla*_DHA_, *bla*_KPC_ and *bla*_NDM_ genes, ABI Prism 3700 DNA sequencer (Applied Biosystems, Foster City, CA, USA) was used for nucleotide sequencing with corresponding primers. The amplicons were sequenced, and molecular type was determined through comparing the DNA sequences to the National Center for Biotechnology Information (NCBI) database at www.ncbi.nlm.nih.gov/blast. 

### 2.6. Identification of Outer Membrane Porins (OmpK35 and OmpK36)

All CRKP isolates were grown in Mueller-Hinton broth to the logarithmic phase and were lysed by sonification. Bacterial outer membrane porins (OMPs) were obtained by the rapid outer membrane protein procedure [27]. Samples were first heated to 100 °C for 5 min, and 12% sodium dodecyl sulphate-polyacrylamide gel electrophoresis (SDS-PAGE) followed by Coomassie blue staining (Bio-Rad, Hercules, CA, USA) was used to identify the OMP profiles (OmpK35 and OmpK36). *K. pneumoniae* ATCC 13883 was used as the control strain [26].

### 2.7. Statistical Analyses

Chi-square tests or Fisher’s exact tests were used to test for statistical significance in comparisons of categorical variables. Two-tailed Student’s *t*-tests or Mann-Whitney U tests were used to test for statistical significance in comparisons of continuous variables. Multivariate Cox proportional hazards regression was used to identify independent risk factors for 14-day and 28-day mortality. We performed an additional subgroup analysis restricted to the critically ill and non-critically ill patients. The results were expressed as hazard ratios (HRs) and 95% confidence intervals (CIs). All biologically plausible variables with *p* ≤ 0.20 in the univariate analysis were tested for collinearity, and linear relationship did not exist between variables. We did two additional analyses to test the statistical sensitivity of the results. In the first sensitivity test, we defined early appropriate antimicrobial therapy as those receiving in vitro active treatments within 5 days of the onset of infection. In the second sensitivity test, we only analyzed patients with signs suggestive of infection, including fever or at least 2 of 4 systemic inflammatory response syndrome (SIRS) criteria: (1) temperature >38 °C or <36 °C; (2) heart rate >90 beats/minute; (3) respiratory rate > 20 breaths/minute or partial pressure of CO_2_ <32 mm Hg; and (4) white blood cell count >12,000/μL, <4000 μL, or >10% immature (band) forms. Data were analyzed using SPSS ver. 17 (SPSS, Chicago, IL, USA). A *p*-value < 0.05 was considered statistically significant.

## 3. Results

### 3.1. Clinical Characteristics of Patients with CRKP Bacteriuria

Overall, 188 patients with CRKP bacteriuria were identified, of whom 62 were excluded. The reasons for exclusion were polymicrobial colonization or infection (*n* = 57), and death within 48 h (*n* = 5). Of the remaining 126 patients, only 107 patients were analyzed for outcomes because 19 patients did not receive any antimicrobial therapy. Fifty-two patients were male, and the mean age was 74.6 ± 14.5 years. Of these patients, 70 (65.4%) had hospital-acquired bacteriuria, 25 (23.4%) had healthcare-associated bacteriuria, and 12 (11.2%) had community-acquired bacteriuria. The majority of enrolled patients (*n* = 76, 71.0%) were urinary catheterized. The 14-day and 28-day mortality of the patients was 14.0% and 25.2%, respectively. The mean APACHE II score was 19.9 ± 9.3. Forty-nine patients (45.8%) with CRKP bacteriuria were defined as critically ill based on an APACHE II score ≥ 20.

Only 33 patients (30.8%) received appropriate antimicrobial therapy. The remaining 74 patients (69.2%) received inappropriate antimicrobial therapy, including 6 patients treated with tigecycline only. Clinical characteristics and outcomes of patients receiving appropriate and inappropriate antimicrobial therapy were similar (Table 1). The detailed antimicrobial regimens were shown in Table S1.

### 3.2. Microbiologic Characteristics of CRKP Isolates

Among 126 CRKP isolates, the MIC ranges, MIC_50_ values, and MIC_90_ values of antimicrobial agents are shown in Table S2. Regarding carbapenem resistance mechanisms, 54 isolates had carbapenemase genes: *bla*_KPC-2_ (*n* = 43), *bla*_KPC-17_ (*n* = 7), *bla*_IMP-8_ (*n* = 2), *bla*_VIM_ (*n* = 1), and *bla*_OXA-48_ (*n* = 1). Among the remaining 72 isolates without carbapenemase genes, 55 isolates had genes encoding ESBLs. Thirty-two of these isolates exhibited the CTX-M-9 group, and 12 isolates exhibited the CTX-M-1 group. Sixteen isolates harbored the SHV-type ESBLs genes, including *bla*_SHV-5_ (*n* = 8), *bla*_SHV-12_ (*n* = 7), and *bla*_SHV-31_ (*n* = 1). Forty-six isolates (63.9%) harbored genes coding for plasmid-borne AmpC, including *bla*_DHA-1_ (*n* = 42) and *bla*_CMY-2_ (*n* = 4). Thirty-eight isolates had two or more ESBL and/or AmpC genes. Regarding the expression of outer membrane porins, 23 isolates lacked OmpK35, two isolates lacked OmpK36, and 43 isolates lacked both OmpK35 and OmpK36. Only four isolates preserved expression of both OmpK35 and OmpK36.

### 3.3. Factors Associated with Mortality

Clinical characteristics were compared between 14-day survivors and non-survivors with CRKP bacteriuria (Table 2). Univariate analysis revealed that APACHE II score ≥ 20 and the presence of shock were associated with 14-day mortality (Table 3). Multivariate Cox regression analysis (Table 3) identified APACHE II score ≥ 20 (HR, 6.15; 95% CI, 1.27–29.73; *p* = 0.024) as the only independent factor associated with 14-day mortality.

Regarding risk factors of the 28-day mortality, univariate analysis revealed that immunocompromised state, APACHE II score ≥ 20 and presence of shock, were significantly associated with 28-day mortality (Table S3). Multivariate Cox regression analysis (Table S3) identified male sex (HR, 2.57; 95% CI, 1.06–6.24; *p* = 0.037) and APACHE II score ≥ 20 (HR, 3.05; 95% CI, 1.21–7.69; *p* = 0.018) as the independent factors for 28-day mortality. Appropriate antimicrobial therapy was not associated with 14-day or 28-day survival.

We applied the early appropriate antimicrobial therapy (within 5 days after index culture) in the sensitivity analysis, and found it was not associated with either 14-day or 28-day survival (results not shown). We further analyzed patients with fever or ≥ 2 of SIRS criteria, but appropriate antimicrobial therapy still could not reduce 14-day or 28-day survival (Tables S4 and S5).

### 3.4. Factors Associated with Mortality in Critically Ill Patients and Non-Critically Ill Patients

Clinical characteristics of critically ill and non-critically ill patients are shown in Table 4. Compared to non-critically ill patients, critically ill patients had a higher proportion of heart failure (46.9% versus 15.5%, *p* < 0.001), cerebrovascular disease (49.0% versus 22.4%, *p* = 0.004), chronic kidney disease (73.5% versus 44.8%, *p* = 0.003), and had a higher mean Charlson comorbidity index (5.10 versus 3.24, *p* < 0.001). Critically ill patients had a significantly higher 14-day mortality (26.5% versus 3.4%, *p* = 0.001) and 28-day mortality (38.8% versus 13.8%, *p* = 0.003) than patients who were not critically ill. In the subgroup analyses restricted to critically ill patients, appropriate antimicrobial therapy was associated with 14-day survival benefit with borderline statistical significance (*p* = 0.055), but it showed no benefit in 28-day mortality (Table 5 and Table 6). In non-critically ill patients, no analysis was performed to identify risk factors for 14-day mortality due to the limited number of events (*n* = 2), and appropriate antimicrobial therapy was not associated with 28-day survival in this subgroup (Table S6).

## 4. Discussion

This study found that antibiotics were commonly used in patients with CRKP bacteriuria (84.9% received any kind of antimicrobial therapy) but appropriate antimicrobial therapy did not have an effect on either 14-day or 28-day mortality. The APACHE II score ≥ 20 was the only independent risk factor for 14-day mortality. Male sex and APACHE II score ≥ 20 were the independent risk factors for 28-day mortality. Among critically ill patients, appropriate antimicrobial therapy was associated with 14-day survival benefit with borderline statistical significance (*p* = 0.055), but it showed no benefit in 28-day mortality. 

The 14-day and 28-day mortality of patients with CRKP bacteriuria was 14.0% and 25.2% in this study. The published outcomes of CRKP bacteriuria have shown a wide range of mortality rates, and the risk factors for death have not been investigated [16,17,18]. Qureshi et al. found a 30-day mortality of 6% among patients with CRKP bacteriuria [18], which is much lower than that found in our study and in other studies [16,17], and no patients died due to CRKP infection. Satlin et al. found a 30-day mortality of 16%, and the 30-day mortality did not differ according to whether patients received any antibiotics [16]. Shilo et al. found that in-hospital mortality of CRKP bacteriuria was 29%. One recent study showed that 30-day mortality was 23.2% in patients with CRKP urinary tract infection [22]. The high mortality rate of patients with CRKP bacteriuria may indicate the severity of their illness and it warrants further study. This is the first study to assess factors associated with death in patients with CRKP bacteriuria, and only APACHE II score and male sex were associated with mortality. This suggests that host factors play a more important role than antimicrobial treatment in the outcome of CRKP bacteriuria.

Babich et al. examined the effect of appropriate empirical antibiotics on survival among catheter-associated urinary tract infections (UTIs) in a single center in Israel, and found that their use was not associated with short-term or long-term survival [28]. A subgroup analysis in patients with bacteremia or septic shock showed similar results [28]. Another multinational retrospective study also found that appropriate empirical antibiotics did not reduce 30-day mortality among patients with complicated UTIs [29]. The authors of the two studies then recommended that physicians consider supportive treatment and monitoring in stable patients with catheter-associated UTIs or complicated UTIs, and withholding antibiotics until the causative pathogen has been defined. 

Our study compared the clinical characteristics and outcomes among critically ill and non-critically ill patients with CRKP bacteriuria. The finding that critically ill patients had a higher 14-day and 28-day mortality suggests that CRKP bacteriuria was not only common in critically ill patients but also a marker of poor prognosis. Appropriate antimicrobial therapy use did not reduce 14-day or 28-day mortality in critically ill patients. We thus propose that patients with CRKP bacteriuria could be completely evaluated clinically and observed without treating them with an antibiotic targeting CRKP in urine. 

This study had several limitations. First, it was a retrospective study, and we did not have information on the clinical symptoms, so we could not differentiate asymptomatic bacteriuria from UTI. We tried to analyze patients with fever or ≥2 SIRS criteria to minimize the possibility of colonization, but we acknowledged that SIRS criteria were not recommended for diagnosing sepsis in the 2016 Surviving Sepsis Guideline [30]. Second, we did not have information about the management of indwelling urinary catheter among these patients, which is an important determinant of treatment outcome. Third, we did not have data of follow-up urine culture results, so we could not assess whether the pathogen cleared. Finally, only 42.9% of the CRKP isolates in our study carried carbapenemase genes. This is lower than in that reported in other studies of CRKP conducted in Western countries [2], which possibly limits the implications of our study findings.

## 5. Conclusions

In patients with CRKP bacteriuria, the APACHE II score ≥ 20 was the only independent factor associated with 14-day mortality. Patients with male sex and APACHE II score ≥ 20 were the independent risk factors for 28-day mortality. However, we found that appropriate antimicrobial therapy was not associated with a lower 14-day or 28-day mortality. Our findings suggest that physicians need to determine the focus of the infection and avoid the overuse of antibiotics in CRKP bacteriuria in patients with less severe illness. Furthermore, a larger prospective study is warranted to assess the role of appropriate antimicrobial therapy on treatment outcomes, especially in critically ill patients.

## Figures and Tables

**Table 1:** Comparison of characteristics and outcomes of patients receiving appropriate antimicrobial therapy and those receiving inappropriate antimicrobial therapy.

Variable	Appropriate Antimicrobial Therapy (*n* = 33)	Inappropriate Antimicrobial Therapy (*n* = 74)	*p* Value
Demographics			
Age, years, mean ± SD	72.06 ± 15.33	75.69 ± 14.02	0.232
Male sex	19 (57.6)	33 (44.6)	0.215
LOS before CRKP isolation, days, mean ± SD	24.73 ± 34.03	25.07 ± 35.45	0.963
Carbapenemase-producing CRKP	14 (42.4)	30 (40.5)	0.855
Previous hospitalization ^1^	15 (45.5)	40 (54.1)	0.411
Comorbidities			
Diabetes mellitus	17 (51.5)	41 (55.4)	0.709
COPD	7 (21.2)	12 (16.2)	0.532
Heart failure	12 (36.4)	20 (27.0)	0.330
Cerebrovascular disease	8 (24.2)	29 (39.2)	0.133
Chronic kidney disease ^2^	20 (60.6)	42 (56.8)	0.710
Liver cirrhosis	2 (6.1)	3 (4.1)	0.643
Malignancy	8 (24.2)	27 (36.5)	0.212
Immunocompromised state	4 (12.1)	8 (10.8)	0.843
Charlson Comorbidity Index, mean ± SD	3.97 ± 2.38	4.15 ± 2.88	0.755
Indwelling urinary catheter	22 (66.7)	54 (73.0)	0.507
Nasogastric tube	21 (63.6)	50 (67.6)	0.691
Surgical drainage	2 (7.5)	7 (9.5)	0.559
Mechanically ventilated at isolation	9 (27.3)	13 (17.6)	0.251
Renal dialysis at isolation	6 (18.2)	5 (6.8)	0.072
APACHE II score, mean ± SD	19.45 ± 10.08	20.11 ± 8.96	0.738
Shock	2 (6.1)	5 (6.8)	0.893
LOS after CRKP isolation, days, mean ± SD	23.17 ± 24.12	23.59 ± 25.43	0.491
14-day mortality	3 (9.1)	12 (16.2)	0.327
28-day mortality	9 (27.3)	18 (24.3)	0.746
all-cause mortality	9 (27.3)	21 (28.4)	0.906

Data are expressed as No. (%) unless otherwise specified. Abbreviations: SD, standard deviation; LOS, length of hospital stay; COPD, chronic obstructive pulmonary disease; APACHE, Acute Physiology and Chronic Health Evaluation. ^1^ During the 3 months preceding infection onset. ^2^ Defined as estimated glomerular filtration rate ≤ 60 mL/min/1.73 m^2^.

**Table 2:** Comparison of characteristics of 14-day survivors and 14-day non-survivors with carbapenem-resistant *Klebsiella pneumoniae* (CRKP) bacteriuria.

Variable	14-Day Survivors (*n* = 92)	14-Day Non-Survivors (*n* = 15)	*p* Value
Demographics			
Age, years, mean ± SD	74.58 ± 14.73	74.53 ± 13.16	0.992
Male sex	44 (47.8)	8 (53.3)	0.692
LOS before CRKP isolation, days, mean ± SD	24.77 ± 35.32	26.13 ± 32.99	0.889
Carbapenemase-producing CRKP	38 (41.3)	6 (40.0)	0.924
Previous hospitalization ^1^	47 (51.1)	8 (53.3)	0.872
Comorbidities			
Diabetes mellitus	46 (50.0)	12 (80.0)	0.031
COPD	15 (16.3)	4 (26.7)	0.330
Heart failure	25 (27.2)	7 (46.7)	0.126
Cerebrovascular disease	31 (33.7)	6 (40.0)	0.634
Chronic kidney disease ^2^	53 (57.6)	9 (60.0)	0.862
Liver cirrhosis	4 (4.3)	1 (6.7)	0.537
Malignancy	30 (32.6)	5 (33.3)	0.956
Immunocompromised state	10 (10.9)	2 (13.3)	0.779
Charlson Comorbidity Index, mean ± SD	3.90 ± 2.67	5.27 ± 2.87	0.072
Indwelling urinary catheter	64 (69.6)	12 (80.0)	0.409
Nasogastric tube	59 (64.1)	12 (80.0)	0.228
Surgical drainage	8 (8.7)	1 (6.7)	0.793
Mechanically ventilated at isolation	19 (20.7)	3 (20.0)	0.954
Renal dialysis at isolation	10 (10.9)	1 (6.7)	0.619
APACHE II score, mean ± SD	18.27 ± 8.13	29.93 ± 9.83	<0.001
Appropriate antimicrobial therapy	30 (32.6)	3 (20.0)	0.327
Shock	4 (4.3)	3 (20.0)	0.023

Data are expressed as No. (%) unless otherwise specified. Abbreviations: SD, standard deviation; LOS, length of hospital stay; COPD, chronic obstructive pulmonary disease; APACHE, Acute Physiology and Chronic Health Evaluation. ^1^ During the 3 months preceding infection onset. ^2^ Defined as estimated glomerular filtration rate ≤ 60 mL/min/1.73 m^2^.

**Table 3:** Univariate and multivariate Cox regression analysis of risk factors for 14-day mortality among patients with CRKP bacteriuria.

Variable	Univariate		Multivariate	
	HR (95% CI)	*p* Value	HR (95% CI)	*p* Value
Age	1.00 (0.96–1.03)	0.887	0.98 (0.94–1.03)	0.415
Male sex	1.17 (0.42–3.23)	0.761	2.41 (0.75–7.80)	0.141
Diabetes mellitus	3.29 (0.93–11.65)	0.065	2.80 (0.70–11.26)	0.146
Heart failure	2.10 (0.76–5.78)	0.153	0.94 (0.29–3.10)	0.924
Charlson Comorbidity Index	1.17 (0.98–1.40)	0.080	1.07 (0.87–1.33)	0.519
APACHE II score ≥ 20	8.00 (1.80–35.43)	0.006	6.15 (1.27–29.73)	0.024
Appropriate antimicrobial therapy	0.49 (0.14–1.74)	0.269	0.57 (0.12–2.63)	0.471
Shock	4.84 (1.36–17.22)	0.015	2.92 (0.70–12.15)	0.140

Abbreviations: APACHE, Acute Physiology and Chronic Health Evaluation.

**Table 4:** Clinical characteristics and outcomes of non-critically ill and critically ill patients with CRKP bacteriuria.

Variable	Non-Critically Ill Patients (APACHI II < 20) (*n* = 58)	Critically Ill Patients (APACHI II ≥ 20) (*n* = 49)	*p* Value
Demographics			
Age, years, mean ± SD	72.79 ± 15.26	76.67 ± 13.30	0.168
Male sex	33 (56.9)	19 (38.8)	0.062
LOS before CRKP isolation, days, mean ± SD	13.38 ± 20.08	38.67 ± 41.02	<0.001
Carbapenemase-producing CRKP	22 (37.9)	22 (44.9)	0.466
Previous hospitalization ^1^	33 (56.9)	22 (44.9)	0.216
Comorbidities			
Diabetes mellitus	28 (48.3)	30 (61.2)	0.180
COPD	13 (22.4)	6 (12.2)	0.170
Heart failure	9 (15.5)	23 (46.9)	<0.001
Cerebrovascular disease	13 (22.4)	24 (49.0)	0.004
Chronic kidney disease ^2^	26 (44.8)	36 (73.5)	0.003
Liver cirrhosis	1 (1.7)	4 (8.2)	0.177
Malignancy	19 (32.8)	16 (32.7)	0.991
Immunocompromised state	7 (12.1)	5 (10.2)	0.761
Charlson Comorbidity Index, mean ± SD	3.24 ± 2.53	5.10 ± 2.62	<0.001
Indwelling urinary catheter	36 (62.1)	40 (81.6)	0.026
Nasogastric tube	27 (46.6)	44 (89.8)	<0.001
Surgical drainage	6 (10.3)	3 (6.1)	0.504
Mechanically ventilated at isolation	3 (5.2)	19 (38.8)	<0.001
Renal dialysis at isolation	1 (1.7)	10 (20.4)	0.002
APACHE II score, mean ± SD	12.98 ± 4.20	28.10 ± 6.52	<0.001
Appropriate antimicrobial therapy	20 (34.5)	13 (26.5)	0.375
Clinical outcomes			
14-day mortality	2 (3.4)	13 (26.5)	0.001
28-day mortality	8 (13.8)	19 (38.8)	0.003
Shock	0 (0.0)	7 (14.3)	0.003

Data are expressed as No. (%) unless otherwise specified. Abbreviations: SD, standard deviation; LOS, length of hospital stay; COPD, chronic obstructive pulmonary disease; APACHE, Acute Physiology and Chronic Health Evaluation. ^1^ During the 3 months preceding infection onset. ^2^ Defined as estimated glomerular filtration rate ≤ 60 mL/min/1.73 m^2^.

**Table 5:** Univariate and multivariate Cox regression analysis of risk factors for 14-day mortality among critically ill patients with CRKP bacteriuria.

Variable	Univariate		Multivariate	
	HR (95% CI)	*p* Value	HR (95% CI)	*p* Value
Age	1.00 (0.96–1.04)	0.946	1.00 (0.95–1.04)	0.892
Male sex	1.31 (0.44–3.91)	0.623	0.69 (0.19–2.60)	0.588
COPD	2.47 (0.67–9.08)	0.173	1.15 (0.28–4.82)	0.845
APACHE II score	1.16 (1.06–1.28)	0.002	1.21 (1.07–1.36)	0.002
Appropriate antimicrobial therapy	0.43 (0.10–1.93)	0.269	0.16 (0.03–1.04)	0.055
Shock	2.42 (0.66–8.83)	0.181	1.65 (0.35–7.88)	0.530

Abbreviations: COPD, chronic obstructive pulmonary disease; APACHE, Acute Physiology and Chronic Health Evaluation.

**Table 6:** Univariate and multivariate Cox regression analysis of risk factors for 28-day mortality among critically ill patients with CRKP bacteriuria.

Variable	Univariate		Multivariate	
	HR (95% CI)	*p* Value	HR (95% CI)	*p* Value
Age	0.98 (0.96–1.01)	0.208	0.99 (0.96–1.02)	0.450
Male sex	1.43 (0.58–3.52)	0.439	1.28 (0.45–3.63)	0.645
APACHE II score	1.14 (1.05–1.24)	0.001	1.13 (1.03–1.23)	0.009
Appropriate antimicrobial therapy	1.06 (0.40–2.78)	0.914	0.61 (0.18–2.05)	0.427
Shock	2.88 (0.92–8.98)	0.068	1.82 (0.46–7.21)	0.396

Abbreviations: APACHE, Acute Physiology and Chronic Health Evaluation.

## Supplementary Materials

The following are available online at https://www.mdpi.com/2076-2607/8/12/2035/s1, Table S1: Mortality of patients according to antimicrobials administered, Table S2: Antimicrobial susceptibility of the CRKP isolates, Table S3: Univariate analysis and multivariate Cox regression analysis of risk factors for 28-day mortality among patients with CRKP bacteriuria, Table S4: Univariate and multivariate Cox regression analysis of risk factors for 14-day mortality among patients with CRKP bacteriuria who had fever or ≥ 2 of SIRS criteria, Table S5: Univariate and multivariate Cox regression analysis of risk factors for 28-day mortality among patients with CRKP bacteriuria who had fever or ≥ 2 of SIRS criteria, Table S6: Univariate and multivariate Cox regression analysis of risk factors for 28-day mortality of non-critically ill patients with CRKP bacteriuria.

## Abbreviations

CRKP: carbapenem-resistant Klebsiella pneumoniae; ESBL: extended spectrum β-lactamase; APACHE: Acute Physiology and Chronic Health Evaluation; National Health Research Institutes (NHRI); MIC: minimal inhibitory concentration; OMP: outer membrane protein; SDS-PAGE: sodium dodecyl sulphate-polyacrylamide gel electrophoresis; HR: hazard ratio; CI: confidence interval; SIRS: systemic inflammatory response syndrome; SD: standard deviation; LOS: length of hospital stay; COPD: chronic obstructive pulmonary disease; UTI: urinary tract infection.
