# Supplementary material for: Does Antimicrobial Therapy Affect Mortality of Patients with Carbapenem-Resistant Klebsiella pneumoniae Bacteriuria? A Nationwide Multicenter Study in Taiwan
Source: Microorganisms. 2020 Dec 19;8(12):2035. doi: 10.3390/microorganisms8122035 (PMC7767250; doi:10.3390/microorganisms8122035)
Supplement: Supplementary file 1 [file microorganisms-08-02035-s001.pdf]

Table S1. Mortality of patients according to antimicrobials administered.

Antimicrobial Regimen	Number of patients			14-Day
	Total	Survived	Died	Mortality (%)
Inappropriate antimicrobial therapy	93	75	12	13.8%
No treatments	19	18	1	5.3%
Antimicrobial regimens not *in vitro* active	68	57	11	16.2%
Tigecycline	6	5	1	16.7%
Appropriate antimicrobial therapy	33	30	3	9.1%
Amikacin/Gentamicin	16	16	0	0.0%
Colistin	10	7	3	30.0%
Carbapenem	1	1	0	0.0%
Ciprofloxacin/Levofloxacin	4	4	0	0.0%
Trimethoprim/sulfamethoxazole	1	1	0	0.0%
Cefepime + Trimethoprim/sulfamethoxazole	1	1	0	0.0%

Table S2. Antimicrobial susceptibility of the CRKP isolates.

Antimicrobial agent	MIC[a] range (μg/mL)	MIC_{50}[b] (μg/mL)	MIC_{90}[c] (μg/mL)	No. (%) of isolates susceptible
Ciprofloxacin	≤0.06 - ≥4	≥4	≥4	7 (5.6)
Levofloxacin	≤0.50 - ≥8	≥8	≥8	12 (9.5)
Piperacillin-tazobactam	16 - ≥128	≥128	≥128	1 (0.8)
Ceftriaxone	≥32	≥32	≥32	0 (0)
Ceftazidime	8 - ≥32	≥32	≥32	2 (1.6)
Cefepime	≤1 - ≥32	≥32	≥32	10 (7.9)
Amikacin	≤4 - ≥64	≤4	≥64	95 (75.4)
Gentamicin	≤1 - ≥16	≤1	≥16	67 (53.2)
Ertapenem	≤0.25 - ≥8	≥8	≥8	3 (2.4)
Imipenem	1 - ≥8	≥8	≥8	2 (1.6)
Meropenem	≤0.25 - ≥8	≥8	≥8	21 (16.7)
Doripenem	≤0.25 - ≥8	≥4	≥8	23 (18.3)
Colistin	≤0.5 - ≥4	≤0.5	≥4	109 (86.5)
Tigecycline	≤0.25 - ≥4	0.5	2	117 (92.9)
Trimethoprim/sulfamethoxazole	≤0.50 - ≥4	≥4	≥4	26 (20.6)

[a]MIC: minimal inhibitory concentration.

[b]MIC_{50}: MIC for 50% of isolates.

[c]MIC_{90}: MIC for 90% of isolates.

Table S3. Univariate and multivariate Cox regression analysis of risk factors of 28-day mortality for patients with CRKP bacteriuria

Variable	Univariate		Multivariate	
	**HR (95% CI)**	**p value**	**HR (95% CI)**	***p* value**
Age	1.00 (0.97–1.02)	0.851	1.00 (0.97–1.02)	0.889
Male sex	1.56 (0.72–3.36)	0.257	2.57 (1.06–6.24)	0.037
Heart failure	1.78 (0.82–3.83)	0.144	1.06 (0.46–2.47)	0.889
Immunocompromised state	2.82 (1.13–7.04)	0.026	2.99 (0.99–8.99)	0.052
APACHE II score ≥20	1.10 (1.05–1.16)	<0.001	3.05 (1.21–7.69)	0.018
Appropriate antimicrobial therapy	0.92 (0.41–2.05)	0.835	0.97 (0.41–2.27)	0.935
Shock	4.82 (1.62–14.30)	0.005	2.27 (0.66–7.81)	0.195

Abbreviations: APACHE, Acute Physiology and Chronic Health Evaluation.

Table S4. Univariate and multivariate Cox regression analysis of risk factors for 14-day mortality among patients with CRKP bacteriuria who had fever or ≥ 2 of SIRS criteria.

Variable	Univariate		Multivariate	
	HR (95% CI)	p value	HR (95% CI)	*p* value
Age	1.00 (0.96–1.03)	0.828	0.99 (0.95–1.04)	0.788
Male sex	1.11 (0.39–3.15)	0.851	2.26 (0.68–7.53)	0.184
Diabetes mellitus	3.97 (0.89–17.73)	0.071	4.48 (0.91–22.12)	0.066
Heart failure	2.13 (0.75–6.07)	0.158	1.31 (0.42–4.06)	0.646
APACHE II score ≥20	4.84 (1.08–21.62)	0.039	3.34 (0.60–18.57)	0.168
Appropriate antimicrobial therapy	0.57 (0.16–2.06)	0.394	0.71 (0.14–3.68)	0.708
Shock	3.86 (1.07–13.88)	0.039	2.94 (0.71–12.26)	0.138

Abbreviations: APACHE, Acute Physiology and Chronic Health Evaluation.

Table S5. Univariate and multivariate Cox regression analysis of risk factors for 28-day mortality among patients with CRKP bacteriuria who had fever or ≥ 2 of SIRS criteria.

**Variable**	**Univariate**		**Multivariate**	
	**HR (95% CI)**	**p value**	**HR (95% CI)**	***p* value**
Age	1.00 (0.96–1.03)	0.828	0.99 (0.95–1.03)	0.478
Male sex	1.11 (0.39–3.15)	0.851	1.45 (0.48–4.36)	0.508
Heart failure	2.13 (0.75–6.07)	0.158	1.32 (0.41–4.24)	0.645
APACHE II score ≥20	4.84 (1.08–21.62)	0.039	3.66 (0.72–18.77)	0.119
Appropriate antimicrobial therapy	0.57 (0.16–2.06)	0.394	0.56 (0.12–2.65)	0.461
Shock	3.86 (1.07–13.88)	0.039	2.54 (0.65–9/90)	0.178

Abbreviations: APACHE, Acute Physiology and Chronic Health Evaluation.

Table S6. Univariate and multivariate Cox regression analysis of risk factors for 28-day mortality of non critically ill patients with CRKP bacteriuria.

Variable	Univariate		Multivariate	
	**HR (95% CI)**	**p value**	**HR (95% CI)**	***p* value**
Age	1.02 (0.97–1.08)	0.372	0.99 (0.94–1.04)	0.656
Male sex	5.69 (0.70–46.24)	0.104	6.52 (0.71–59.69)	0.097
Immunocompromised state	11.08 (2.21–55.46)	0.003	12.33 (2.08–73.15)	0.006
Appropriate antimicrobial therapy	0.92 (0.22–3.85)	0.909	0.86 (0.20–3.78)	0.839
